# High Frequency 10 kHz Spinal Cord Stimulation as a First Line Programming Option for Patients With Chronic Pain: A Retrospective Study and Review of the Current Evidence

**DOI:** 10.7759/cureus.17220

**Published:** 2021-08-16

**Authors:** Mickey E Abraham, Justin Gold, Akhil Dondapati, Kristin Sheaffer, Julian L Gendreau, Antonios Mammis

**Affiliations:** 1 Neurological Surgery, University of California, La Jolla, USA; 2 Department of Neurosurgery, Rutgers New Jersey Medical School, Newark, USA; 3 Department of Neurosurgery, Rutger New Jersey Medical School, Newark, USA; 4 Orthopedic Surgery, Mercer University School of Medicine, Savannah, USA; 5 Department of Biomedical Engineering, Johns Hopkins Whiting School of Engineering, Baltimore, USA

**Keywords:** back pain, chronic pain, high frequency, spinal cord, stimulation, 10 khz

## Abstract

Introduction

Neuromodulation is an evolving and increasingly popular therapy for chronic pain management. Recent data suggest that novel waveforms have demonstrated greater benefit over traditional spinal cord stimulation (SCS). The authors conducted a retrospective review of patients undergoing high-frequency 10 kHz SCS at a single tertiary medical center for the purpose of contributing further evidence to this growing body of data. The literature of high-frequency SCS published to date was also reviewed.

Methods

A retrospective chart review was performed for patients with chronic pain syndrome, including failed back surgery syndrome and sciatica alone, who underwent high-frequency SCS at 10 kHz. This data was analyzed using R software (R Foundation for Statistical Computing, Vienna, Austria) for statistical analysis. The PubMed database was searched for relevant articles using the search terms “high frequency,” “10 kHz,” and “spinal cord stimulation.” All relevant studies conducted to date were included in this literature review.

Results

Twenty-one patients had complete follow-up data and were included in this study. Of the 21 patients, 85.7% subjectively reported post-operative pain relief while 71.4% of the total patients reported pain relief by ≥ 50%. There was a statistically significant decrease in mean VAS scores from pre-operative to 12-months post-operative (8.52 vs 4.37, p < 0.001). Additionally, 76.5% of patients subjectively reported improvements in sleep and activities of daily living. Recent studies indicate that high-frequency SCS appears to be a viable option for delivering quality pain relief in patients for chronic regional pain syndrome, failed back surgery syndrome, sciatica, and also pain in the upper cervical region of the spine.

Conclusion

This article provides evidence both with the authors’ own institutional data and from the currently published literature for the efficacy of using high-frequency SCS at 10 kHz as a first-line programming option for patients undergoing SCS.

## Introduction

Chronic pain is a widely prevalent and debilitating health condition with a total financial cost reported to range from $560 - $635 billion [1-3]. Neuromodulation is currently an evolving and increasingly popular therapy for chronic pain management [4]. Spinal cord stimulation (SCS), first introduced in 1967, is an established modality for long-term pain management. A significant limitation of traditional SCS is that its low-frequency stimulations elicit sensations of paresthesia over a patient’s targeted pain distribution [5].

A recent major advancement has been the ability to modulate higher frequency and faster waveforms for SCS, which has shown diverse levels of effectiveness in controlling chronic pain without eliciting the sensations of paresthesia that lower frequency waveforms can create [4,6,7]. Thus, this high-frequency SCS at 10 kHz (HF10-SCS) could prove to be a superior method of targeting residual low back pain [5,8,9]. While the precise mechanism of the HF10-SCS’s inability to not elicit sensations of paresthesia remains unclear, computational modeling suggests that small and medium-diameter fibers are recruited for the desired pain modulation while large-diameter axons carrying vibratory sensation are preferentially blocked [10]. Other hypotheses suggest its ability to block depolarization signals, desynchronize neuronal signals, disrupt membrane integration, disrupt glial-neuronal interaction and inhibit spinal mitogen-activated protein kinases could be involved [11,12].

Therefore, the authors conducted a retrospective review of their institutional records for patients implanted with HF10-SCS as a first-line stimulation programming option in patients who have not undergone traditional SCS. Through this review of a single surgeon experience, pain relief, changes in sleep, changes in the use of pain medications, and changes in activities of daily living were measured and statistically analyzed. The literature on high-frequency spinal cord stimulation published to date was also reviewed.

## Materials and methods

Patient selection

A retrospective chart review was performed using data for patients with chronic pain syndrome, including failed back surgery syndrome (FBSS) and sciatica, who underwent HF10-SCS implantation. All procedures were performed between February 2018 and February 2019, with all patients having been actively treated for at least 12 months with medical management prior to the implant of the SCS. Prior to the SCS implantation, a pain physician evaluated the patient to determine the medical appropriateness of the procedure. Meetings with each of the patients were made with the psychologist, pain physician, and nurse practitioner to determine goals of treatment, including functional goals and medication reduction targets. After a thorough multidisciplinary evaluation, patients fulfilling the criteria for SCS implantation participated in a trial evaluation period for around five to seven days. Subjects with a successful trial, defined as at least 50% pain relief, proceeded to permanent implant with the Senza Omnia SCS system (Nevro Corp., Menlo, CA). Only patients who previously underwent spine surgery were described as having FBSS; all the other patients did not have prior spine surgeries.

Surgical technique

One surgeon performed all of the procedures with the same surgical technique. The patient was positioned prone on a Wilson frame, and the patient was placed under local anesthesia with conscious sedation. An incision was made two levels below the location of the caudal contact leads. Soft-tissue dissection was performed with midline laminotomy using Kerrison rongeurs and Leksell rongeurs as needed. Leads were placed according to the directions for use of the system, and programming resulted in therapy without paresthesia. Ten kHz was set to a pulse width of 30 μs and an average amplitude of 22qdwwq1111q.88 (+/- 0.15) mA. After placement of the spinal cord stimulator, the incision was closed with layered sutures. 

Data analysis

Patients were requested to follow up at three, six, nine, and 12 months to monitor progress in terms of pain relief via VAS score, use of pain medication, activities of daily living, and quality of sleep. Descriptive statistics, including the mean, median, standard deviation (SD), and interquartile range (IQR) was used to describe differences between baseline and 12-month postoperative VAS scores in the patient population. If the patient reported a different VAS score from the leg compared to the back, the mean of both scores was calculated. All analyses were carried out with R software (R Foundation for Statistical Computing, Vienna, Austria). 

Literature search

The PubMed database was searched for relevant articles using the search terms “high frequency,” “10 kHz,” and “spinal cord stimulation.” All relevant prospective and retrospective studies conducted to date were included in this literature review.

## Results

Twenty-one patients had complete follow up data and were included in this study. Demographics are displayed in Table 1. All patients had a 12-month VAS score. Of these 21 patients included in the patient series, 17 of the patients (81.0%) included information about changes in sleep and activities of daily living, and 16 of the patients (76.2%) offered information about pain medication use.

**Table 1:** Demographics of patients undergoing high frequency spinal cord stimulation at 10kHz included in the study

Gender	
Female	11
Male	10
Age (years)	
Mean ± SD	59.5 ± 12.3

There were no significant intraoperative or perioperative complications. Of the 21 patients, 85.7% reported pain relief after SCS implantation and 71.4% reported pain relief by ≥50% relief by 12 months (Table 2). The mean pain relief of the group was a reduction by 58.3% (SD 31.0 [95% CI 44.2 - 72.4]). The mean difference in starting VAS score and 12-month VAS score was 4.15; a variable that achieved statistical significance (8.52 vs 4.37, p < 0.001).

**Table 2:** Clinical outcomes of pain scores, changes in sleep, changes in activity and changes in pain medication usage VAS - visual analog scale

Patient Number	Age	Sex	Diagnosis	Pain Distribution	Starting Average VAS	12-Month Average VAS	Average Difference VAS	Change in Sleep	Change in Activity	Change in Pain Medication Use
1	36	F	Failed Back Surgery Syndrome	Back	7	2	4.5	Improved	Improved	Decreased
				Bilateral LE	7	3				
2	55	F	Failed Back Surgery Syndrome	Back	7	7	0	Improved	Improved	Decreased
				Bilateral LE	7	7				
3	74	M	Failed Back Surgery Syndrome	Back	8.5	1	7.75	Improved	Improved	Decreased
				Bilateral LE	9	1				
4	38	F	Failed Back Surgery Syndrome	Back	10	1	9	Not improved	Not improved	Same
5	45	M	Failed Back Surgery Syndrome	Back	5	2.5	2.5	-	-	-
				Bilateral LE	5	2.5				
6	56	F	Failed Back Surgery Syndrome	Back	9	2.5	6.75	Improved	Improved	Same
				Bilateral LE	8	1				
7	65	F	Failed Back Surgery Syndrome	LLE	7	0	7	Improved	Improved	Same
8	62	M	Failed Back Surgery Syndrome	Bilateral LE	10	10	0	Not improved	Not improved	-
9	67	M	Failed Back Surgery Syndrome	Back	8	5	3	-	Improved	-
10	75	F	Failed Back Surgery Syndrome	Back	9	5	4	Improved	-	-
11	50	M	Failed Back Surgery Syndrome	Back	7.5	5	2.5	-	-	-
12	58	F	Failed Back Surgery Syndrome	Back	10	1	9	Improved	Improved	Decreased
13	60	M	Failed Back Surgery Syndrome	Back	10	10	0	Not improved	Not improved	Increased
14	83	M	Failed Back Surgery Syndrome	Back	10	3	7	-	-	Same
15	57	F	Failed Back Surgery Syndrome	Back	10	5	5	Improved	Improved	Same
16	55	F	Failed Back Surgery Syndrome	Back	6	5	1	Improved	Improved	Same
				Bilateral LE	6	5				
17	46	M	Failed Back Surgery Syndrome	Back	9	4	5	Improved	Improved	Decreased
18	70	F	Sciatica	L leg	10	3	7	Improved	Improved	Decreased
				L foot	10	3				
19	61	M	Sciatica	Back	8.5	6	2.75	Improved	Improved	Same
				LLE	9	6				
20	62	F	Sciatica	Back	9.5	6	3.5	Improved	Improved	Decreased
21	75	M	Sciatica	Back	8	8	0	Not improved	Not improved	Same
All	-	-	-	-	8.52 (95% CI 7.85-9.20)	4.37 (95% CI 3.07-5.66)	4.15 (p < 0.001)	-	-	-

Improvements to functionality were also observed by 12 months, with 76.5% (13/17) of the patients reporting improvement in sleep and activities of daily living. Of the 16 patients that reported pain medication use, 43.8% (7/16), 50% (8/16), and 6.3% (1/16) of them indicated decreased, same, and increased pain medication use, respectively. Decreased pain medication use is described as any patient that has a decreased dosage from their preoperative regimen.

## Discussion

Clinically meaningful, long-term pain relief at 12-months without adverse events was achieved with HF10-SCS in 85.7% of the patient population reported in this retrospective review. The findings of this study are consistent with what has been shown with previously conducted prospective and retrospective studies, but it also further demonstrates the feasibility of utilizing HF10-SCS as a first-line programming option over traditional waveforms [13]. 

Review of the current evidence

Low Back Pain

The first evidence for the use of HF10-SCS for the treatment of chronic pain was in 2013, when Van Buyten et al. published a prospective multicenter European clinical study of patients with difficult-to-treat chronic back pain. They showed that HF10-SCS provided significant and sustained low back pain relief to more than 70% of treated subjects six months postoperatively [14]. In 2014, Al-Kaisy et al. further examined these patients and reported findings of decreased low back pain at 24 months post-implantation with an additional decrease in patient-perceived disability and opioid use [15]. 

Later in 2015, a United States multicenter, randomized, controlled trial demonstrated the superiority of HF10-SCS for low back pain and reported an opioid dose reduction at three and 12 months post-implantation compared to the traditional low-frequency SCS [16]. In 2016, the same authors published a follow-up study at 24 months and reported further improvements in pain, self-reported disability, and overall patient satisfaction [13]. 

In Australia, a 2016 study by Russo et al. published the collective experience of centers that were using HF10-SCS since the technology received regulatory approval from its national government [17]. A total of 256 patients with back pain who failed low-frequency SCS and/or peripheral nerve field stimulation, trialed HF10-SCS. Of these, 189 (73%) reported a positive trial and were implanted. A mean reduction of a >50% pain score was sustained for up to 6 months and a mean 7 point reduction in Oswestry Disability Index (ODI) was observed.

In 2019, Stauss et al. designed a large observational, multicenter, retrospective review of the effectiveness of HF10-SCS. Approximately a quarter of the patients in this study had failed traditional SCS [18]. 1,660 patients were included in the review and over 70% of patients reported response to therapy throughout the 12 months follow-up period. Most of their patients also reported concomitant improvements in activities of daily living (72.3%), sleep (68.0%), and quality of life (90.3%). Further, 32% of patients reported decreased pain medication use at the final follow-up. This was the largest review to provide complementary evidence to support the treatment of chronic back pain with HF10-SCS. 

Chronic Regional Pain Syndrome

In 2018, Gill et al. retrospectively reviewed data from all patients with CRPS at one center that underwent HF10-SCS trial and permanent implantation [19]. Eight of the 13 patients who underwent implantation had previously experienced inadequate benefit from traditional SCS and the remaining five patients had never attempted a prior stimulation. Their small case series suggests that HF10-SCS may be a viable option for patients with CRPS including those who had initially experienced suboptimal results from traditional SCS.

In addition, Ghosh et al. in 2020 retrospectively reviewed all CRPS and failed back surgery syndrome (FBSS) patients who failed traditional SCS trials in a tertiary care center [20]. Herein, 31 patients met the criteria and trialed HF10-SCS, of these, 28 (90%) underwent permanent implantation. Overall, 46.4% were excellent responders with an average reduction of 63.7% in pain scores at a median follow-up of 21 months. 

Upper Extremity

In 2019, Salmon et al. reported long-term (2.3 +/- 1.6 years) outcomes of patients receiving HF10-SCS for the treatment of combined upper and lower body neuropathic pain syndromes [21]. A total of 45 patients underwent a trial of combined cervical and thoracic HF10-SCS leads placed over C2/T2 or C2/T9 vertebral levels, or three leads placed over C2/T2/T9 levels. Around 38 patients (84.4%) reported a successful trial and were permanently implanted. Likewise, a reduction in both disability and opioid pain medication use was observed. Over 90% of patients were satisfied with their treatment outcome.

With all of this currently published evidence, the use of HF10-SCS appears to be a viable option for providing adequate pain relief in patients that have failed traditional SCS frequencies. Additionally, it also appears to be a viable alternative to traditional lower-frequency stimulation programmings and should be considered for use as a first-line programming option for patients experiencing low back pain, CRPS, and upper extremity pain.

Limitations

The major limitation of this study is that it is a retrospective single-center investigation, which included only 21 patients. While this study lacks the scientific robustness of a prospective controlled trial, it serves an important purpose of providing further evidence of pain reduction in patients with chronic pain syndromes who undergo treatment with HF10-SCS as a first-line stimulation programming treatment option. 

## Conclusions

This study was designed to evaluate the effectiveness of HF10-SCS as a first-line programming treatment modality in patients with chronic pain syndromes, including FBSS and sciatica who had not undergone initial therapy with traditional SCS beforehand. This retrospective investigation demonstrated that the therapy provided was effective and resulted in sustained pain relief at 12 months for an overwhelming majority of the patients (85.7%), with most of the patients also having greater than 50% pain relief (71.4%). Therefore, this article provides further evidence, from both the authors' own institutional data and currently published literature, for the efficacy of beginning to use HF10-SCS as a first-line programming option for patients undergoing spinal cord stimulation.
